# Tropical Oil Consumption and Cardiovascular Disease: An Umbrella Review of Systematic Reviews and Meta Analyses

**DOI:** 10.3390/nu13051549

**Published:** 2021-05-04

**Authors:** Chanita Unhapipatpong, Prapimporn Chattranukulchai Shantavasinkul, Vijj Kasemsup, Sukanya Siriyotha, Daruneewan Warodomwichit, Sirikan Maneesuwannarat, Prin Vathesatogkit, Piyamitr Sritara, Ammarin Thakkinstian

**Affiliations:** 1 Division of Nutrition and Biochemical Medicine, Department of Medicine, Faculty of Medicine Ramathibodi Hospital, Mahidol University, Bangkok 10400, Thailand; chanita@kkumail.com (C.U.); daruneewanw@gmail.com (D.W.); 2 Graduate Program in Nutrition, Faculty of Medicine Ramathibodi Hospital, Mahidol University, Bangkok 10400, Thailand; 3 Department of Community Medicine, Faculty of Medicine Ramathibodi Hospital, Mahidol University, Bangkok 10400, Thailand; vijj9@hotmail.com; 4 Department of Clinical Epidemiology and Biostatistics, Faculty of Medicine Ramathibodi Hospital, Mahidol University, Bangkok 10400, Thailand; sukanya.sii@mahidol.edu (S.S.); ammarin.tha@mahidol.edu (A.T.); 5 Fat Consumption Project, Faculty of Medicine Ramathibodi Hospital, Mahidol University, Bangkok 10400, Thailand; season085@gmail.com; 6 Division of Cardiology, Department of Medicine, Faculty of Medicine Ramathibodi Hospital, Mahidol University, Bangkok 10400, Thailand; prin.vat@mahidol.ac.th (P.V.); piyamitr.sri@mahidol.ac.th (P.S.)

**Keywords:** tropical oil, vegetable oil, palm oil, coconut oil, lard, soybean oil, rice bran oil, cardiovascular disease, lipid

## Abstract

The health effects of saturated fat, particularly tropical oil, on cardiovascular disease are unclear. We investigated the effect of tropical oil (palm and coconut oils), lard, and other common vegetable oils (soybean and rice bran oils) that are widely used in tropical and Asian countries on lipid profiles. We performed an umbrella review of meta-analyses and systematic reviews. Electronic databases (Medline, Scopus, Embase, and Cochrane) were searched up to December 2018 without language restriction. We identified nine meta-analyses that investigated the effect of dietary oils on lipid levels. Replacement of polyunsaturated fatty-acid-rich oils (PUFAs) and monounsaturated FA-rich oils (MUFAs) with palm oil significantly increased low-density lipoprotein cholesterol (LDL-c), by 3.43 (0.44–6.41) mg/dL and 9.18 (6.90–11.45) mg/dL, respectively, and high-density lipoprotein cholesterol (HDL-c), by 1.89 (1.23–2.55) mg/dL and 0.94 (−0.07–1.97) mg/dL, respectively. Replacement of PUFAs with coconut oil significantly increased HDL-c and total cholesterol –by 2.27 (0.93–3.6) mg/dL and 5.88 (0.21–11.55) mg/dL, respectively—but not LDL-c. Substituting lard for MUFAs and PUFAs increased LDL-c–by 8.39 (2.83–13.95) mg/dL and 9.85 (6.06–13.65) mg/dL, respectively—but not HDL-c. Soybean oil substituted for other PUFAs had no effect on lipid levels, while rice bran oil substitution decreased LDL-c. Our findings show the deleterious effect of saturated fats from animal sources on lipid profiles. Replacement of unsaturated plant-derived fats with plant-derived saturated fats slightly increases LDL-c but also increases HDL-c, which in turn may exert a neutral effect on cardiovascular health.

## 1. Introduction

Cardiovascular disease (CVD) is responsible for the largest proportion of deaths worldwide [1], and dyslipidemia is an important modifiable risk factor for the development of CVD [2,3,4]. One non-pharmacologic intervention that can reduce the risk of CVD is the modification of dietary fat, whereby a reduction in the intake of saturated fats (SFs) and replacement with unsaturated fat may reduce the risk of CVD. Several studies have shown that high intake of SFs increases low-density lipoprotein cholesterol (LDL-c) level [5]. However, there is some evidence to indicate that consumption of SFs, particularly tropical oils (e.g., coconut and palm oils), might not increase the risk of CVD [6,7]. According to the United States Department of Agriculture (USDA), the most commonly consumed oils worldwide are palm oil and soybean oil [8]. Lard, coconut oil, and rice bran oil (RBO) are also popular in tropical region and throughout Asia. Based on their major FA components, palm oil, coconut oil, and lard are classified as SFs, while soybean oil and RBO are classified as polyunsaturated fatty acid (PUFA)-rich oil and monounsaturated fatty acid (MUFA)-rich oil, respectively. Palm oil and coconut oil are commonly used oils in tropical countries and both contain high amounts of SFs, which can increase LDL-cholesterol and may increase the risk of CVD. However, the potential benefits of coconut oil, which also contains medium chain triglycerides (MCT), have led to an increase in its popularity. Palm oil is unique in that it contains high amounts of SFs and MUFAs, and has increasingly been used as an alternative to partially hydrogenated fats [9]. Lard is an animal-derived fat that has received much attention in the popular media because of its positioning as a naturally sourced, heat-stable cooking fat which contains both SFAs and MUFAs. Although dietary guidelines generally recommend restricting the intake of SFs, there is no clear consensus on the health effects of SFs from current evidence, particularly with respect to the effects of oils from tropical regions on CVD. We, therefore, conducted an umbrella review to systemically assess the existing evidence and evaluate the effect on lipid parameters of tropical oil (palm oil and coconut oil), lard, and other common vegetable oils (soybean oil and RBO) that are widely used in Asian and tropical countries.

## 2. Materials and Methods

### 2.1. Literature Search and Selection Criteria

The study protocol was registered in the international prospective register of systematic reviews (PROSPERO; CRD42019130581). We conducted a review of multiple systematic reviews (SR) and meta-analyses (MAs) in compliance with standardized procedures [10,11]. Relevant SRs and MAs were identified from electronic databases (MEDLINE/PubMed, Scopus, Embase, and Cochrane central register of controlled trials (CENTRAL)) up to December 2018 without any language restriction using the search terms and search strategies described in Supplementary Data S1.

### 2.2. Eligibility Criteria

Studies were selected independently by two authors (C.U. and P.C.S.) and disagreements were resolved through consensus with a third author (V.K.). Studies were eligible if they met all of the following criteria: SRs and MAs of randomized controlled trials (RCT) or observational studies; inclusion of adult patients; comparison of coconut oil, palm oil, RBO, soybean oil, or lard as a dietary intervention with any other edible oils on iso-caloric exchange; and reporting of any lipid outcomes (LDL-c, HDL-c, triglyceride, total cholesterol (TC), or TC-to-HDL-c ratio). SRs or MAs that evaluated oils in the form of dietary supplements or drugs or the postprandial effect of oil intake on blood lipids were excluded.

### 2.3. Data Extraction

One reviewer (C.U.) extracted the following data for each study: first author, year of publication, origin (country), number and type of included studies (*N*), number of participant (*n*), sex, mean age, body mass index (BMI, kg/m^2^), baseline TC, study population, dietary interventions and comparators and duration (days), outcomes, pooling method, effect size (ES, e.g., mean difference (MD)), 95% confidence interval (95% CI), conflicts of interest, and funding source. The senior reviewer (A.T.) evaluated and verified the extracted data, and any disagreement was solved by consensus.

### 2.4. Quality Assessment

Methodological quality was independently assessed by two reviewers (C.U. and P.C.S.) using the AMSTAR2 tool [12]. Grading was classified into critically low (more than one critical flaw with or without non-critical weaknesses), low (one critical flaw with or without non-critical weakness), moderate (more than one non-critical weakness), or high (no more than one non-critical weakness) confidence. Disagreements were resolved by consensus between authors.

### 2.5. Data Analysis

Characteristics of MAs (e.g., setting, type of included studies, *N*, *n*) and their findings (ESs and 95% CIs) were described. An overlap of primary included studies was estimated across included MAs using a corrected covered area (CCA) [13]. CCA was classified as slight overlap, moderate, high, or very high if the percent overlap was 0–5%, 6–10%, 11–14%, or >15%, respectively. Data from individual included RCTs/cohorts were extracted, and ESs along with their variances were estimated and re-pooled using a random-effect model. Heterogeneity was assessed using Cochran’s Q test and Higgin’s I² statistic, and was deemed present for a *p* value < 0.1 or *I*^2^ ≥ 50% [14]. All analyses were performed using STATA version 16.0 (StataCorp. 2019. Stata Statistical Software: Release 16. College Station, TX: StataCorp LLC)].

## 3. Results

**Figure 1:**
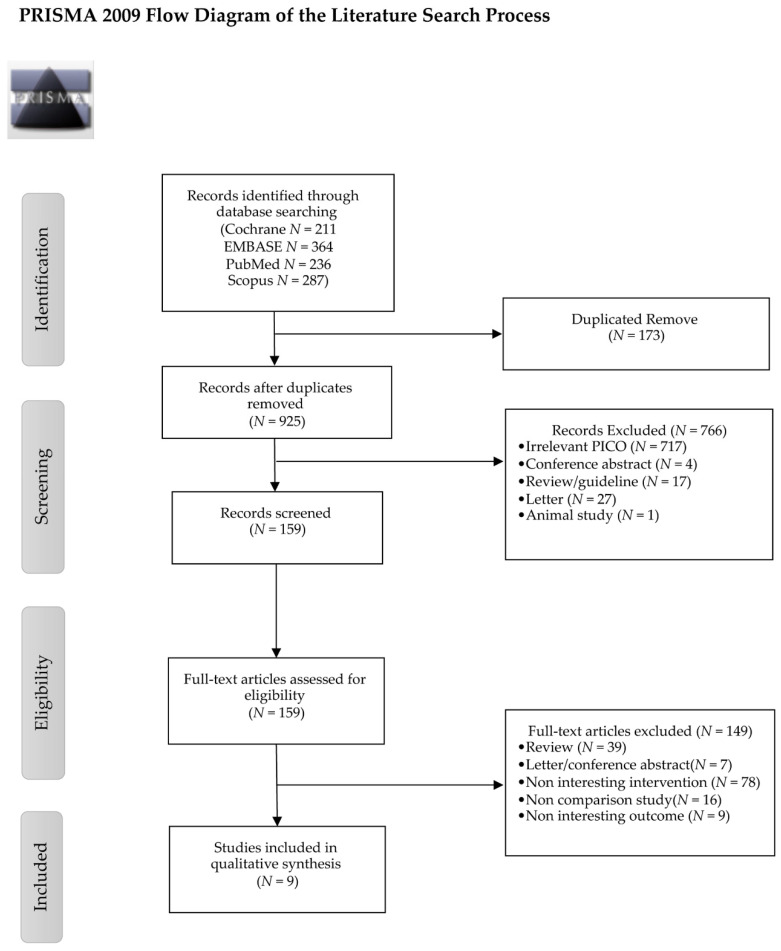
PRISMA 2009 flow diagram of the literature search process.

A total of 1098 studies were identified, of which 9 met the inclusion criteria (Figure 1) [15,16,17,18,19,20,21,22,23].

The characteristics of the included studies are summarized in Table 1 and Supplementary Table S2. The degree of overlap of included primary studies on lipid outcome was assessed (Supplementary Table S3), and the resulting CCA of 7.4% indicated moderate overlap among the primary studies. The AMSTAR2 assessment results are summarized in Supplementary Table S4 for lipid outcomes and Supplementary Table S5 for clinical outcomes. Among the 9 studies with fasting lipid outcomes, the number of included primary studies ranged from 2 to 51, with sample sizes of 34 to 2065 (Table 1). The studies were published between 2009 and 2018 and had study durations of 2–27 weeks. Mean ages and BMI were 16–84 years and 17–37.4 kg/m^2^, respectively. The included populations varied from healthy individuals to patients with CVD risk factors or established CVD. For all studies, intervention oils were replaced with comparator oils in an isocaloric fashion. Indirect comparison from network meta-analysis [23] is also included in the present review. Where there was more than one comparison, the largest study was selected for the present review and indirect comparison of the effect of individual oils was shown on the forest plot with an asterisk (*). Re-pooling of ESs on lipid markers is described in the following sections.

### 3.1. LDL–c Outcome

**Figure 2:**
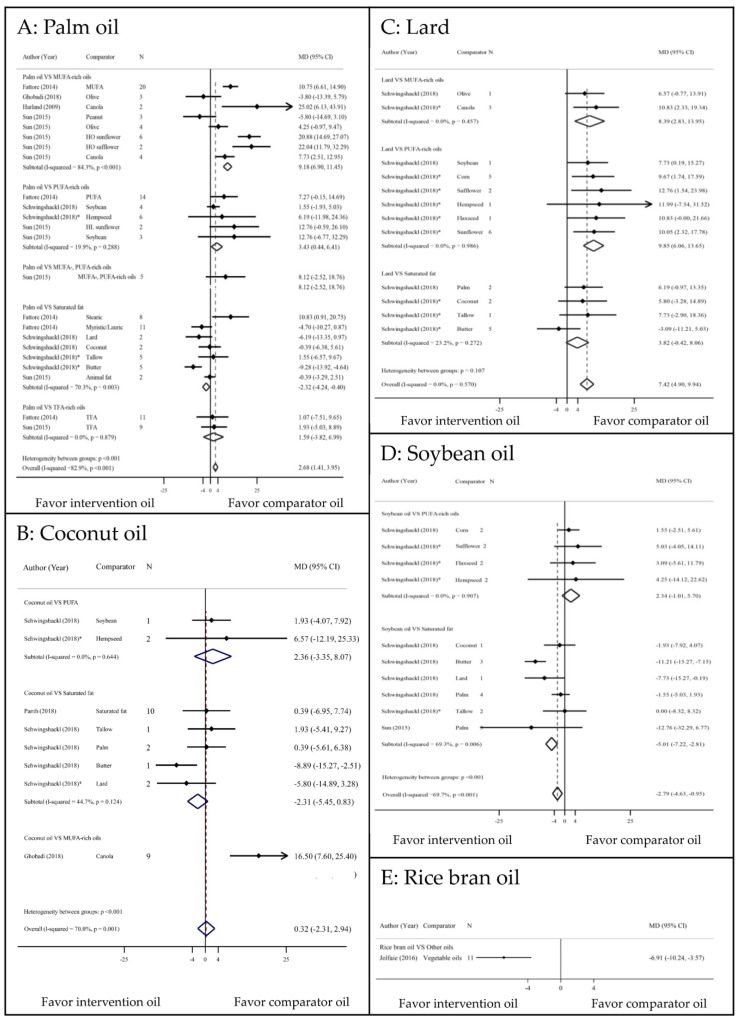
Comparisons of LDL–c levels among PUFA, MUFA, and SFAs. CI: confidence interval; HL: high linoleic; HO: high oleic; MD: mean difference; MUFA: monounsaturated fatty acid; PUFA: polyunsaturated fatty acid; TFA: trans fatty acid; * indirect comparison.

ESs of all LDL-c comparisons are displayed in Figure 2. Palm oil significantly increased LDL-c concentration compared with MUFA and PUFA with the corresponding MDs (95% CIs, I^2^) of 9.18 mg/dL (6.90 to 11.45, 84.3%) and 3.43 mg/dL (0.44 to 6.41, 19.9%) (Figure 2a). In addition, palm oil was associated with a significantly lower LDL-c than those of other SFs, with MDs of −2.32 (−4.24 to −0.40, 70.3%), except for trans-fatty acid (TFA) rich oils. Coconut oil increased LDL-c compared with PUFA, although this effect was not significant (Figure 2b). Conversely, coconut oil was associated with a lower LDL-c than other SFs, though this difference was not statistically significant. Compared with MUFA- and PUFA-rich oils, lard was associated with significant increases in LDL-c (8.39 mg/dL increase; 2.83 to 13.95, 0% and 9.85 mg/dL increase; 6.06 to 13.65, 0%, respectively). Lard also caused higher LDL-c levels than other SFs, although this difference was not significant (3.82 mg/dL increase; −0.42 to 8.06, 23.2%) (Figure 2c). Soybean oil caused an insignificant increase in LDL-c compared with PUFA-rich oils (2.34 mg/dL increase; −1.01 to 5.7, 0%) [23]. Conversely, soybean oil was associated with a 5.01 mg/dL decrease in LDL-c (−7.22 to −2.81, 69.3%) compared with other SFs (Figure 2d). Only one study indicated that RBO significantly decreased LDL-c compared with other vegetable oils (−6.91 mg/dL decrease; −10.24 to −3.57) [19] (Figure 2e).

### 3.2. Total Cholesterol Outcome

**Figure 3:**
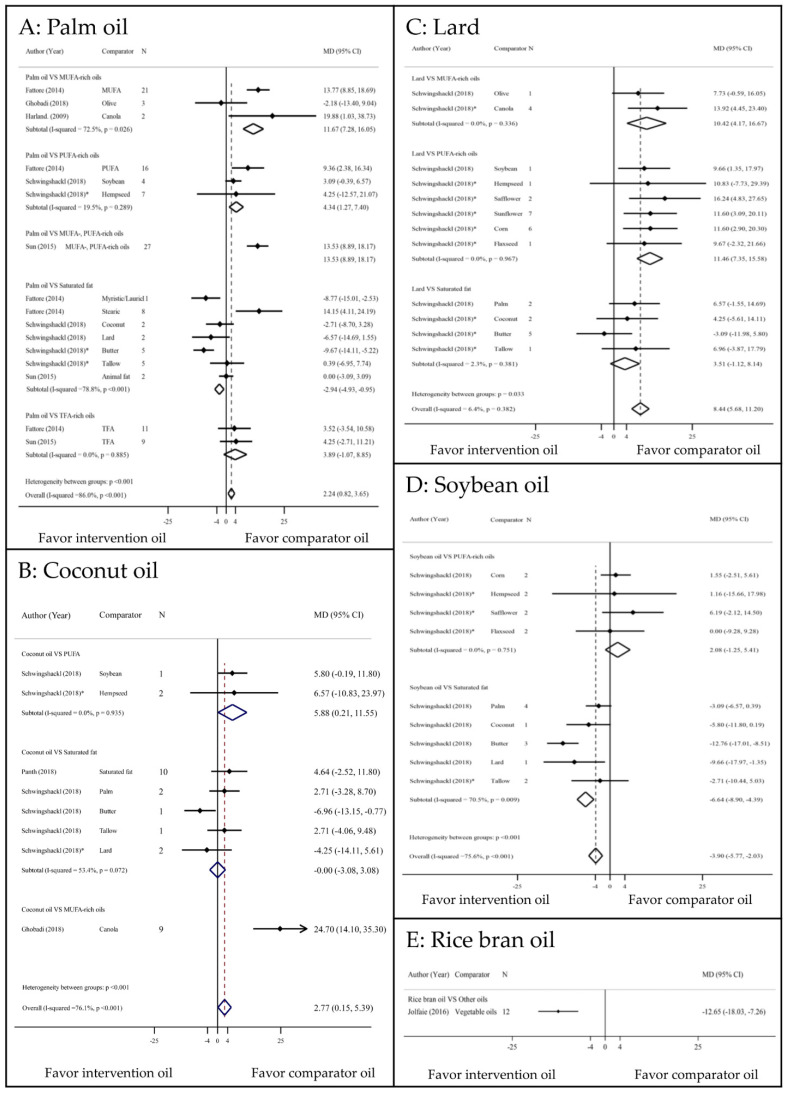
Comparisons of TC levels among PUFA, MUFA, and SFAs. CI: confidence interval; MD: mean difference; MUFA: monounsaturated fatty acid; PUFA: polyunsaturated fatty acid; TFA: trans fatty acid; * indirect comparison.

Compared with MUFA and PUFA-rich oils, palm oil significantly increased TC (11.67 mg/dL increase; 7.28 to 16.05, 72.5% and 4.34 mg/dL increase; 1.27 to 7.4, 19.5%, respectively) (Figure 3a). Conversely, palm oil caused decrease TC of −2.94 (−4.93 to −0.95, 78.8%) which was significantly lower than those of other SFs. Coconut oil had a significantly increased TC of 5.88 mg/dL (0.21 to 11.55, 0%) compared with PUFA, although this difference was not significant compared with other SFs (Figure 3b). Other SFs (palm oil, coconut oil, and dairy products) significantly increased TC of 24.7 mg/dL (14.1 to 35.3) [20] compared with canola oil. Lard significantly increased TC of 10.42 mg/dL (4.17 to 16.67, 0%) and 11.46 mg/dL (7.35 to 15.58, 0%) compared with MUFA- and PUFA-rich oils, respectively. Lard also had higher TC than other SFs, but this was not significant. Soybean oil significantly decreased TC by −6.64 mg/dL (−8.9 to −4.39, 70.5%) compared with SFs, but there was no difference compared with PUFA-rich oils [23]. Similar to the LDL-c outcome, RBO significantly decreased TC by 12.65 (−18.03 to −7.26) [19] (Figure 3e).

### 3.3. HDL–c Outcome

**Figure 4:**
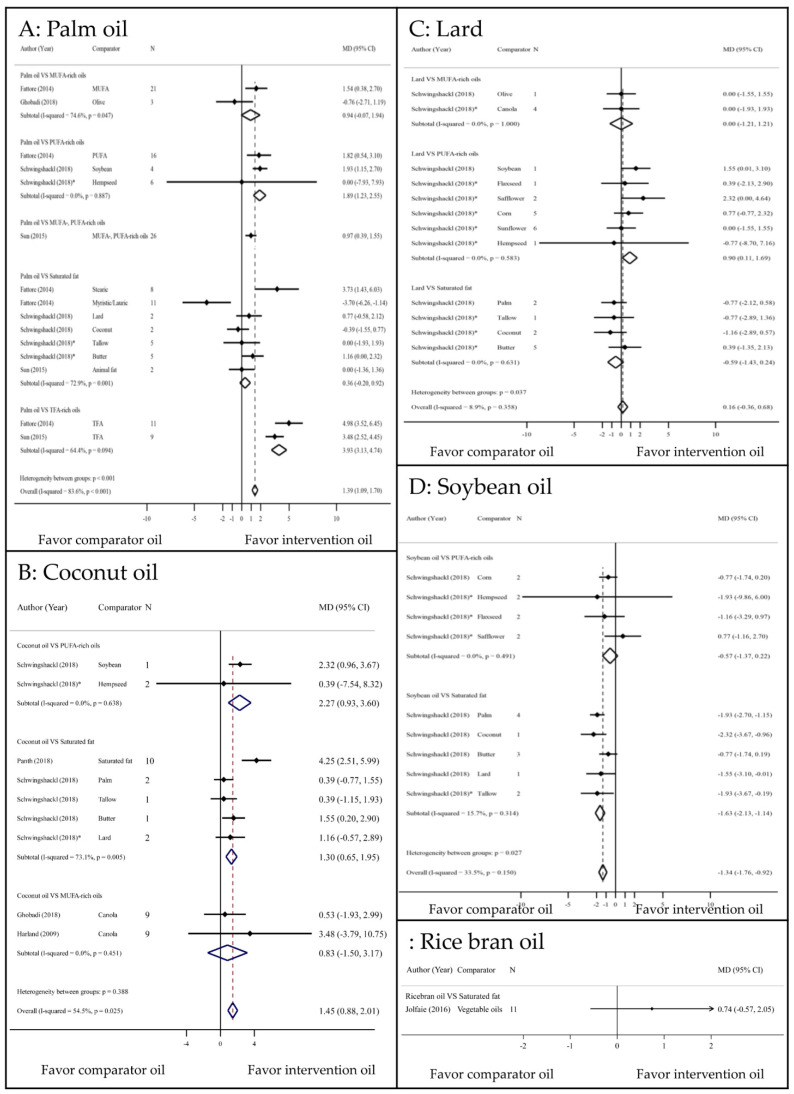
Comparisons of HDL–c levels among PUFA, MUFA, and SFAs. CI: confidence interval; MD: mean difference; MUFA: monounsaturated fatty acid; PUFA: polyunsaturated fatty acid; TFA: trans fatty acid; * indirect comparison.

Palm oil significantly increased HDL-c levels compared with PUFA and TFA-rich oils–by 1.89 mg/dL (1.23 to 2.55, 0%) and 3.93 mg/dL (3.13–4.74, 64.4%), respectively—but there was no significantly difference compared with MUFA-rich oils and other SFs (Figure 4a). In addition, compared with PUFA-rich oils and other SFs, coconut oil significantly increased HDL-c (2.27 mg/dL increase; 0.93 to 3.6, 0% and 1.3 mg/dL increase; 0.65 to 1.95, 73.1%), respectively (Figure 4b). Replacement of butter [23] with coconut oil significantly increased HDL-c (1.55 mg/dL increase; 0.2 to 2.9) (Figure 4b). Lard increased HDL-c compared with PUFA-rich oils–by 0.90 mg/dL (0.11 to 1.69, 0%)—but not MUFA-rich oils and other SFs. Overall, palm oil and coconut oil significantly increased HDL compared with comparator oils; however, lard had no significant effect on HDL (Figure 4c). Soybean oil significantly lowered HDL-c compared with SFs—by −1.63 mg/dL (−2.13 to −1.14, 15.7%)—but not PUFA-rich oils (Figure 4d). RBO had no effect on HDL-c compared with other vegetable oils (Figure 4e) [19].

### 3.4. Triacylglycerol (TAG) Outcome

**Figure 5:**
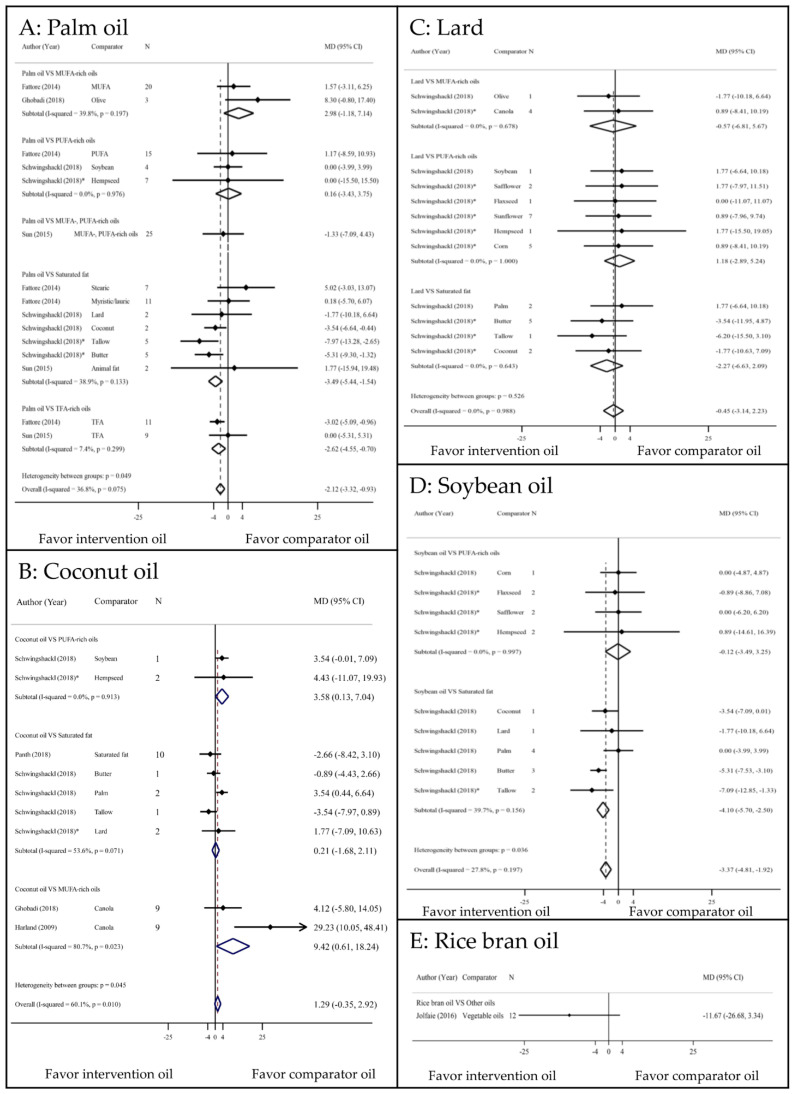
Comparisons of TAG levels among PUFA, MUFA, and SFAs. CI: confidence interval; MD: mean difference; MUFA: monounsaturated fatty acid; PUFA: polyunsaturated fatty acid; TFA: trans fatty acid; * indirect comparison.

Palm oils had higher TAG levels than MUFAs and PUFAs, but the differences were not significant (Figure 5a). However, palm oils had −3.49 (−5.44 to −1.54, 38.9%) and −2.62 (−4.55 to −0.70, 7.4%) mg/dL lower TAG levels than SFs and TFA-rich oils, respectively. Replacement of PUFA-rich oils with coconut oil significantly increased the TAG level (3.58 mg/dL increase; 0.13 to 7.04, 0%), although replacement of other SFs with coconut oil had no effect on TAG levels (Figure 5b). Soybean oil reduced the TAG level by −4.10 mg/dL (−5.70 to −2.50, 39.7%) compared with SFs (Figure 5d) [23]. When lard and RBO [19] were substituted for other oils, no significant effect on TAG levels was observed (Figure 5c,e).

### 3.5. Total Cholesterol to HDL-c Ratio Outcome

In the current study, palm oil and RBO [19] had no significant effect on the TC/HDL-c ratio when substituted for MUFA- or PUFA-rich oil or other SFs. However, palm oil substituted for TFA-rich oil consumption significantly decreased the TC/HDL-c ratio [16,17]. Substituting TFA-rich oils with palm oil, lard, and soybean oil also decreased the TC/HDL-c ratio (Table 2).

## 4. Discussion

We performed an umbrella review to summarize the findings of previous SRs and MAs that explored the association of dietary fat intake with lipid profiles and CVD. Our study confirmed the deleterious effects of SFs and showed that SFs derived from animal and plant sources had different effects on lipid profiles. Although the use of palm oil and coconut oil in place of MUFA- and PUFA-rich oils increased LDL-c and TC (for palm oil) and increased TC (for coconut oil), both oils significantly increased HDL-c. Furthermore, substituting lard for comparator oils increased LDL-c and TC, but not HDL-c. Several studies [24,25,26] have indicated that not all SFs have equal effects on lipid profiles, with differences especially clear between animal-derived and plant-derived fats. Our study supported the current recommendations to reduce dietary SF intake, particularly animal-derived fat, and to replace this source of fat with foods rich in unsaturated FAs from plants to lower the risk of CVD [27].

Previous dietary guidelines from 1980 recommending the limitation of dietary fat intake to less than 30% of total calories were later revised in 2005 to 20–35% of calories with a suggestion to reduce SF intake [28]. However, the prevalence of obesity, diabetes, and CVD has risen substantially despite a reduction in dietary fat intake [29]. In 2015, the Dietary Guidelines Advisory Committee [30] removed the upper limit on dietary fat intake and instead focused on the type of dietary fat. Several studies have indicated that MUFA derived from plant (e.g., olive oil) or animal (e.g., lard) sources is not equivalent to PUFA with respect to the effect on CVD [24,25,26]. The 2019 American College of Cardiology (ACC) and American Heart Association (AHA) guideline on the primary prevention of CVD recommended the replacement of SFs with dietary MUFA- and PUFA-rich oils [31] and also recommended plant-based diets, which are associated with lower mortality than animal-based diets [32,33]. This guidance is similar to that issued by the European Society of Cardiology (ESC) [34]. In tropical countries, palm oil, coconut oil, and lard, categorized as SFs, have been widely used for centuries [35], and the effect of SFs from tropical oils on CVD thus remains controversial [36].

Palm oil is among the most widely used plant-derived SFs in many countries [37], and of its major FA constituent is palmitic acid (C16:0) (45%) [38]. Although other vegetable oils are available, palm oil is inexpensive and therefore affordable for the majority of the population within developing countries [39] and in the food industry. We demonstrated that the replacement of PUFA- and MUFA-rich oils with palm oil significantly increased LDL-c and HDL-c levels. Furthermore, our findings confirmed the deleterious effect of TFA-rich oils, even compared with SFs [17,18]. Palm oil substituted for TFA-rich oils not only decreased TAG and TC/HDL-c ratio but also increased HDL-c level. Compared with coconut oil, palm oil had no effect on LDL-c, TC, or HDL-c, while one study reported that substituting palm oil for coconut oil decreased TAG [23]. Conversely, one MA demonstrated that substituting palm oil for butter, an animal-derived fat, significantly decreased TC, LDL-c, and TAG [23]. This result may be attributable to the large proportion of palmitic acid in palm oil, which is primarily esterified to the terminal carbons of the triglyceride glycerol (sn-1 and sn-3), thus limiting its absorption and its excretion primarily in feces as calcium salts compared with other SFs [40,41].

Coconut oil has been heavily promoted as a healthy oil with benefits for cardiovascular outcomes. It is rich in SFs (82%), including lauric acid (47%) and myristic acid (16.5%). Our study demonstrated that replacement of PUFA-rich oils with coconut oil significantly increased TC and HDL-c but not LDL-c, while coconut oil substituted for other SFs significantly increased HDL-c. Moreover, replacement of butter with coconut oil significantly decreased LDL-c and TC and increased HDL-c. Myristic acid (C14:0) is the most potent cholesterol-raising SF followed by palmitic acid and lauric acid [42]. A major source of myristic acid is dairy fat, which is found in butter and milk [43]. These results emphasized the different effects of animal- and plant-derived SF on lipid profiles. Coconut oil mainly contains lauric acid (C12:0), which behaves predominantly like a long-chain FA from a metabolic standpoint [44]. Thus, research and health claims on MCTs cannot be applied to coconut oil because most FAs in coconut oil differ from MCTs in their structure, absorption, and metabolism [44]. The effect of lauric acid on blood lipids is an increase in both TC and HDL-c, with a greater effect on HDL-c. Thus, lauric-acid rich oils may decrease the ratio of TC to HDL-c [16]. However, no prospective studies or RCTs to date have specifically assessed the effect of coconut oil on CVD outcomes and mortality. Observational data have indicated that indigenous populations consuming coconut as their staple food have a low incidence of CVD [45]; nevertheless, the diets of these populations did not contain high proportions of SFs. Moreover, these populations consumed coconut flesh or squeezed coconut as part of a traditional diet, rather than coconut oil.

Lard is popular in Thai and other Asian cuisines as a cooking oil for fried or deep-fried food. Even though palm oil and lard are rich in MUFA (containing 37% and 45% MUFA, respectively) [36] and lard contains stearic acid (C18:0) (11%), which has no cholesterol-raising effect [46], our study shows that lard did not increase HDL-c compared with comparator oils. Moreover, our findings indicate that substituting lard for MUFAs and PUFAs increased LDL-c and TC. A recent study demonstrated that MUFA intake from plants but not from animals was associated with lower CHD risk [24]. Therefore, consumption of SF-rich oil, particularly animal-derived fat, should be limited to decrease the risk of CVD.

We combined the effect of lipid profile change after substitution of SF with MUFA and PUFA by estimating the 10-year atherosclerotic cardiovascular disease (ASCVD) risk (http://tools.acc.org/ASCVD-Risk-Estimator-Plus/, accessed on 5 January 2020). The result revealed that substituting lard for PUFA and MUFA could increase the 10-year ASCVD risk by approximately 1% in patients with high CV risk (diabetes, hypertension, and smoking). Similarly, substituting palm oil for MUFA-rich oil increased the 10-year ASCVD risk by approximately 1%. However, substituting palm oil for PUFA and substituting coconut oil for PUFA and MUFA did not increase the ASCVD risk. Thus, it may be appropriate to recommend the intake of plant-derived SFs either from palm oil or coconut oil instead of those from animal-derived SFs for health-related outcomes [31].

Soybean oil is the second most commonly consumed oil worldwide [8]. In our analysis, replacement of SF with soybean oil significantly improved all lipid parameters. Substituting soybean oil for lard and butter significantly decreased LDL-c. However, substituting soybean oil for other PUFA-rich oils had no effect on lipid levels.

RBO is a rich source of MUFA (44%), PUFA (33.6%), tocopherols, tocotrienols, and phytosterol [47], and is thus a popular cooking oil in Asia and tropical countries. RBO also contains γ-oryzanol, which has antioxidant properties [48] and has been shown to improve lipid profiles [49,50,51]. Our study findings indicate the favorable effect of RBO in decreasing both LDL-c and TC levels. Our umbrella review included only one MA of the effect of RBO on lipid levels [19].

Our study had some strengths, including the umbrella review design, which is an efficient approach to summarize comprehensive evidence from SRs and MAs. SFAs, PUFAs, and MUFAs from animal and plant sources were included, and their effects on all lipid profiles were pooled. Limitations of our study included variation in dosages of intervention oils and baseline diets across the studies, meaning that most SRs and MAs had substantially heterogeneous findings. Most MAs evaluating groups of oils, such as SFs, PUFAs, and MUFAs, with unidentified individual oils were excluded. Further research into the effect of consumption of tropical oils compared with other common vegetable oils on the number and size of LDL and HDL particles, CVD incidence, and mortality is therefore warranted.

Our study findings support current recommendations to reduce animal-derived SF intake and replace it with soybean oil, RBO, or other PUFA- and MUFA-rich vegetable oils to improve lipid profiles and reduce the risk of CVD. However, the health impact of plant-derived SFs, which are widely used in Asia, remains inconclusive because both ‘good’ and ‘bad’ cholesterol are elevated following their consumption. Animal-derived (lard and butter) and plant-derived (palm and coconut oil) SFs have different effects on lipid profiles. Future guidelines should therefore address this issue and give specific recommendations.

## Figures and Tables

**Table 1:** Baseline characteristics of included systematic reviews and meta-analyses.

Author, Year	Country	Number of Included Studies	*n*	% Male	Mean Age (years)	Intervention Oil	Comparator Oil	Duration of Intervention (days)	Outcome	Baseline Serum Total Cholesterol (mg/dL)	Conflict of Interest Funding Source	AMSTAR2
Harland et al., 2009 [15]	UK	2	34	52.9	18–78	Mixed saturated fat Palm oil (22 g/d) (*30%–39% of total calorie intake from fat)	Canola oil (23 g/d)	18–56	LDL-c, TC, HDL-c, TAG	170 to 275	CI: Food industry organizations FS: Dow AgroSciences, Hertfordshire, UK.	Critically low
Mozaffarian et al., 2009 [16]	USA	13 for lipid parameters	528	53	25–63	Palm oil Lard Soybean oil (* 7.5% of total calorie intake from fat)	20%Trans fatty acid PHO 35% Trans fatty acid PHO 45% Trans fatty acid PHO	17–70	TC/HDL-c, Lap(a)	116 to 278	CI: no conflict of interest FS: University of Otago Research and Enterprise Unit.	Critically low
		4 prospective cohorts for clinical outcomes	139,836	43.6	30–84 (min to max)			5–20 years	Adjusted risk reduction in coronary heart disease (nonfatal myocardial infarction or CHD death)	190 to 282		Critically low
Fattore et al., 2014 [17]	Italy	51	1526	66	16–75	Palm oil (*4%–43% of total calorie intake from fat)	Stearic acid Myristic/lauric oil MUFA PUFA Trans fatty acid PHO Interestified palm oil	14–112	LDL-c, TC, HDL-c, TAG, VLDL-c, apo B, apo A-I, TC/HDL-c, LDL-c/HDL-c, Lap(a)	108 to 271	CI: no conflict of interest FS: Universita Bocconi, Soremartec Italia s.r.l.	Critically low
Sun et al., 2015 [18]	Singapore	32	1073	65.4	16–68	Palm oil (*12%–43% of total energy intake from fat)	Vegetable oil low in saturated fat Trans fat-containing oil Animal fat	14–112	LDL-c, TC, HDL-c, TAG	120 to 341	CI: no conflict of interest FS: The National Medical Research Council, Singapore	Low
Jolfaie et al., 2016 [19]	Iran	11	344	36	34–61	Rice bran oil	Other oils	21–90	LDL-c, TC, HDL-c, TAG, VLDL-c, apo B, apo A, TC/HDL-c, LDL-c/HDL-c, Lap(a)	134 to 325	CI: no conflict of interest FS: NR	Low
Ghobadi et al., 2018 [20]	Iran	9	292	49.31	22–65	Saturated fat (*7%–20% of total energy intake from fat)	Canola oil (12–50 g/d)	21–180	LDL-c, TC, HDL-c, TAG, apo B, apo A-I, LDL/HDL, TC/HDL	130 to 309	CI: no conflict of interest FS: no funding source	Moderate
Ghobadi et al., 2018 [21]	Iran	3	198	58.08	23–84	Palm oil (*3%–81% of total energy intake from fat)	Olive oil (25–60 g/d)	21–180	LDL-c, TC, HDL-c, TAG, apo B, apo A-I	167 to 257	CI: no conflict of interest FS: Shiraz University of Medical Sciences	High
Panth et al., 2018 [22]	Australia	10	299	53.5	21–66	Naturally occurring medium chain fatty acid (*14.2–108 g/d)	Long-chain fatty acid	21–42	LDL-c, TC, HDL-c, TAG, VLDL-c apo A-I, apo B	113 to 274	CI: no conflict of interest FS: no funding source	High
Schwingshackl et al., 2018 [23]	Germany	28	2065	54	22–84	Soy oil, palm oil, coconut oil, lard	Other oils and solid fat	21–189	LDL-c, TC, HDL-c, TAG	130 to 274	CI: no conflict of interest FS: no funding source	High

Apo A-1: Apolipoprotein A-1; Apo B: Apolipoprotein B; CI: Conflict of interest; FS: Funding source; HDL-c: High-density lipoprotein-cholesterol; Lap (a): Lipoprotein (a); LDL-c: Low-density lipoprotein-cholesterol; MUFA: Monounsaturated fatty acid; NA: not reported; NIDDM: Non-insulin dependent diabetes mellitus; PHO: Partially hydrogenated oil; PUFA: Polyunsaturated fatty acid; TAG: Triacylglycerol; TC: Total cholesterol; TFA: Trans fatty acid.

**Table 2:** Ratio of total cholesterol to HDL-cholesterol.

Studies	Intervention	Comparator	*n*	Effect Size (95%CI)	Heterogeneity I^2^ (%)
Fattore et al., 2014	Palm oil	MUFA-rich oils	5	0.02 (−0.1, 0.14)	0.00%
Harland et al.,2009		Canola oil	2	0.77	
Fattore et al., 2014	Palm oil	PUFA-rich oils	5	−0.19 (−0.43, 0.06)	22.71%
Fattore et al., 2014	Palm oil	Stearic acid	3	−0.12 (−0.4, 0.16)	18.43%
Fattore et al., 2014	Palm oil	TFA	3	−0.45 (−0.58, −0.31)	0.00%
Mozaffarian et al., 2009		20% TFA PHO	13	−0.02	
Mozaffarian et al., 2009		35% TFA PHO	13	−0.1	
Mozaffarian et al., 2009		45% TFA PHO	13	−0.14	
Ghobadi et al., 2018 [20]	Palm oil, animal fat	Canola oil	8	0.07 (−0.15, 0.3)	23.2%
Mozaffarian et al., 2009	Lard	20% TFA PHO	13	−0.02	
Mozaffarian et al., 2009		35% TFA PHO	13	−0.09	
Mozaffarian et al., 2009		45% TFA PHO	13	−0.14	
Mozaffarian et al., 2009	Soybean oil	20% TFA PHO	13	−0.12	
Mozaffarian et al., 2009		35% TFA PHO	13	−0.20	
Mozaffarian et al., 2009		45% TFA PHO	13	−0.25	
Jolfaie et al., 2016	Rice bran oil	Vegetable oils	4	−0.08 (−0.22, 0.07)	13%

## Supplementary Materials

The following are available online at https://www.mdpi.com/article/10.3390/nu13051549/s1, Supplementary Data S1: Search Strategy, Table S2: Additional baseline included systematic reviews and meta-analyses, Table S3: Degree of Overlapping Individual Studies in LDL Outcome, Table S4: AMSTARII of Studies with Lipid Outcomes.
