# Supplementary material for: Tropical Oil Consumption and Cardiovascular Disease: An Umbrella Review of Systematic Reviews and Meta Analyses
Source: Nutrients. 2021 May 4;13(5):1549. doi: 10.3390/nu13051549 (PMC8148021; doi:10.3390/nu13051549)
Supplement: Supplementary file 1 [file nutrients-13-01549-s001.zip › nutrients-1179937-supplementary.pdf]

Tropical Oil Consumption and Cardiovascular Disease: an Umbrella Review of Systematic Reviews and Meta analyses

Chanita Unhapipatpong, M.D.

Online Supplementary Material

Supplementary Data S1: Search Strategy

Database: PubMed MEDLINE up to 31th December 2018

1. "Coconut Oil"[MeSH] OR "Coconut Oil" OR "Oil, Coconut" OR "coconut fat" OR "lauric acid"
2. "Palm Oil"[MeSH] OR "palm oil" OR "Elaeis guineensis" OR "Elaeis guinensis" OR "palmolein" OR "palmitic" OR "palmoleic" OR "myristic acid"
3. "rice bran oil" OR "ricebran oil" OR "rice bran wax" OR "rice bran extract" OR "rice oil" OR "rice germ oil" OR "oryza sativa oil" OR "oleic"
4. "soy bean oil" OR "soyabean oil" OR "soybean oil" OR "soya-bean oil" OR "soya oil" OR "Fatty Acids, Omega-6"[MeSH] OR "Linoleic" OR "n 6 fatty acid" OR "omega 6 fatty acid" OR "omega6"[tiab] OR "n-6"[tiab]
5. "lard"[Supplementary Concept] OR "lard" OR "pork lard" OR "solid fat" OR "dietary fat" OR "animal fat" OR "saturated fat" OR "stearic" OR "stearic"[tiab] OR "stearate"[tiab]
6. "plant oil" OR "plant oils" OR "vegetable oil" OR "vegetable oils"
7. #1 OR #2 OR #3 OR #4 OR #5 OR #6
8. "lipid*"[MeSH] OR "lipoprotein*"[tiab] OR "cholesterol*"[MeSH] OR "LDL"[tiab] OR "HDL"[tiab] OR "triglyceride"[tiab] OR "high density"[tiab] OR "low density"[tiab] OR "triacylglycerol*"[tiab] OR "Dyslipidemias"[MeSH] OR "dyslipidemias" OR "dyslipidemia" OR "dyslipidaemia" OR "dyslipoproteinemia"
9. (cardiovascular AND (death OR mortality)) OR "myocardial infarct*" OR "coronary infarct*" OR "heart infarct*" OR "heart attack" OR STEMI OR NSTEMI OR "coronary syndrome" OR (''cardiovascular diseases''[Mesh] OR ''cardiovascular disease'' [tiab] OR ''cardiovascular diseases''[tiab] OR ''heart disease'' [tiab] OR ''heart diseases''[tiab] OR ''myocardial infarction'' [tiab] OR ''myocardial infarctions''[tiab] OR ''heart attack''[tiab] OR ''heart attacks''[tiab] OR ''sudden death''[tiab] OR ''sudden deaths''[tiab] OR ''coronary syndrome''[tiab] OR "cerebrovascular accident" OR CVA OR TIA OR "transient ischemic attack" OR "cerebrovascular disease" OR "cerebral vascular" OR ''stroke''[tiab] OR ''strokes''[tiab] OR ''cerebrovascular accident''[tiab] OR ''cerebrovascular accidents''[tiab]
10. #8 OR #9
11. "Systematic review" OR "meta-analysis" OR "meta-analyses" OR "network meta"
12. #7 AND #10 AND #11

Supplementary Data S1: Search Strategy(Continued)

Database: Ovid Embase upto 31th December 2018

1. 'coconut oil'/exp OR coconut OR 'cocos nucifera extract'/exp OR 'cocos nucifera extract' OR ‘virgin coconut oil’/exp OR ‘virgin coconut oil’ OR ‘lauric acid’/exp OR ‘lauric acid’
2. 'elaeis guineensis'/exp OR 'elaeis guineensis' OR ‘palm oil’/exp OR 'palm oil' OR ‘palm’/exp OR ‘palm’ OR ‘palm kernel oil’/exp OR ‘palm kernel oil’ OR ‘palmolein’/exp OR ‘palmolein’ OR ‘palmitic’/exp OR ‘palmitic’ OR ‘palmoleic’/exp OR ‘palmoleic’ OR ‘myristic acid’/exp OR ‘myristic acid’
3. ‘soybean oil’/exp OR ‘soybean oil’ OR ‘soyabean oil’/exp OR ‘soyabean oil’ OR ‘soy bean oil’/exp OR ‘soy bean oil’ OR ‘soya-bean oil’/exp OR ‘soya-bean oil’ OR ‘soya oil’/exp OR ‘soya oil’ OR ‘linoleic/exp or linoleic OR ‘omega 6 fatty acid’/exp OR ‘omega 6 fatty acid’
4. ‘Rice Bran oil’/exp OR ‘Rice Bran oil’ OR ‘ricebran oil’/exp OR ‘ricebran oil’ OR ‘rice bran extract’/exp OR ‘rice bran extract’ OR ‘rice bran wax’/exp OR ‘rice bran wax’ OR ‘rice bran extract’/exp OR ‘rice oil’/exp OR ‘rice oil’ OR ‘rice germ oil’/exp OR ‘rice germ oil’ OR ‘oryza stiva oil’/exp OR ‘oryza stiva oil’ OR ‘oleic’/exp OR ‘oleic’
5. (Vegetable AND oil) OR ‘dietary fat’/exp OR ‘dietary fat’ OR ‘plant oil’/exp OR ‘plant oil’ OR ‘fat intake’/exp OR ‘fat intake’
6. ‘Lard’/exp OR ‘lard’ OR ‘animal fat’/exp OR ‘animal fat’ OR ‘saturated fat’/exp OR ‘saturated fat’ OR ‘stearic’/exp OR ‘stearic’
7. #1 OR #2 OR #3 OR #4 OR #5 OR #6
8. 'cholesterol'/exp OR 'cholesterol' OR 'dyslipidemia'/exp OR 'dyslipidemia' OR 'lipoprotein'/exp OR 'lipoprotein' OR 'lipid'/exp OR 'lipid' OR 'triacylglycerol'/exp OR 'triacylglycerol' OR 'triglyceride'/exp OR 'triglyceride' OR ‘hypertriglyceridemia’/exp OR ‘hypertriglyceridemia’
9. 'cardiovascular'/exp OR cardiovascular OR ‘cardiovascular system’/exp OR ‘cardiovascular system’ OR ‘‘ischemic heart disease’/exp OR ‘‘ischemic heart disease’ OR ‘cardiovascular death’/exp OR ‘cardiovascular death’ OR ‘death’/exp OR ‘death’ OR ‘mortality’/exp OR ‘mortality’ OR ‘cerebrovascular accident’/exp OR ‘cerebrovascular accident’ OR CVA/exp OR CVA OR stroke/exp OR stroke OR ‘brain infarction’/exp OR ‘brain infarction’
10. #8 OR #9
11. AND ([cochrane review]/lim OR [systematic review]/lim OR [meta analysis]/lim)
12. #7 AND #10 AND #11 13. #12 AND [humans]/lim NOT 2019:py NOT ‘nonhuman’/de

Supplementary Table S2: Additional baseline included systematic reviews and meta-analyses

Author, year	End search	Populations	BMI (kg/m^2)	N	Pooling method	Intervention oil	Comparator oil
Harland et al., 2009 [15]	December 2008	Healthy	21.9-28.4	10 studies (6 crossover)<br>2 RCT as subgroup	Fixed-effects models	Saturated fatty acid (mixed fat, lard, tallow, butter, palm oil)<br>Palm oil (22g/d) as subgroup<br>(*30 to 39% of total calories intake from fat)	-Canola oil (23g/d)
Mozaffarian et al., 2009 [16]	January 2008	Healthy, hypertension obese, hyperlipidemia, cardiovascular history	17.4-36	13 randomized trial (12 crossover and 1 parallel)	Random-effects models	Palm oil<br>Lard<br>Soybean oil	-20%Trans fatty acid PHO<br>-35% Trans fatty acid PHO<br>-45% Trans fatty acid PHO
Fattore et al., 2014 [17]	30 May 2013	Healthy, hyperlipidemia, hyperfibrinogen, NIDDM	17-36	51 studies (45 crossover, 5 parallel, 1 sequential)	Fixed-effects models<br>Random-effects models	Palm oil<br>(*4 to 43% of total calories intake from fat)	-Stearic acid (12-13% equal to tallow, lard, butter)<br>-Myristic/lauric oil (synthetic oil, oil blend, coconut oil)<br>-MUFA (HO safflower oil, HO sunflower oil, olive oil, canola oil, America Health Association blend (50%soybeanoil, 40%palmoil,10% canola oil))<br>-PUFA (HL safflower oil, HL sunflower oil, soybean oil, groundnut oil, peanut oil, soft margarine)<br>-Partially hydrogenated trans fatty acid (margarines)<br>-Interestified palm oil (palm oil esterified in sn-1,3positions, modified palm oil esterified in sn-2)
Sun et al., 2015 [18]	30 May 2014	Healthy, hyperlipidemia, coronary heart disease	17-36	30 articles with 32 studies (28 crossovers, 4 parallel)	Random-effects models	Palm oil<br>(*12% to 43% of total energy intake from fat)	-Vegetable oil low in saturated fat (HO sunflower oil, olive oil, peanut oil, canola oil + sunflower oil, soybean + RBO, soybean + canola oil, HO + HL safflower oil)<br>-Trans fat-containing (Trans blend, partially hydrogenated soybean oil, partially hydrogenated canola oil, margarine)<br>-Animal fat (Lard, sweet butter)
Jolfaie et al., 2016 [19]	30 November 2015	Healthy, hyperlipidemia, NIDDM	21.9-37.4	11 studies (7 crossover, 3 parallel, 1 before-after)	Fixed-effects models (subgroup heterogeneity)<br>Random-effects models (overall effect)	Rice bran oil	Soybean oil (18 g/d), sunflower oil with standard spread, sunflower oil(29g/d), standard spread(20g/d), oil blend with fatty acid composition similar to RBO, peanut oil(50g/d), corn oil, palm oil and peanut oil(39g/d), low-calorie diet without RBO

Ghobadi et al., 2018 [20]	December 2017	Healthy, hyperlipidemia, NAFLD, NIDDM, heart disease, obesity, metabolic syndrome	19.6-31.6	27 studies (15 parallel, 12 crossover)	Random-effects models	Saturated fat (dairy fat, palm oil, coconut oil)<br><br>(*7% to 20% of total energy intake from fat)	-Canola oil (12 -50 g/d)
Ghobadi et al., 2018 [21]	September 2017	Healthy, hyperlipidemia, NAFLD, metabolic syndrome, peripheral vascular disease, rheumatoid arthritis	20.4-32.1	27 studies (13 crossovers, 14 parallel)	Random-effects models	Palm oil<br><br>(*3 to 81% of total energy intake from fat)	-Olive oil (virgin olive oil, refined olive oil) (25-60 g/d)
Panth et al., 2018 [22]	Before 1 April 2018	Healthy, hyperlipidemia	19-29.7	12 studies (11 crossover and 1 parallel)<br>*10 studies for naturally occurring MCFAs<br>*2 studies for manufactured (re-esterified) MCFAs	Random-effects models	Naturally occurring medium chain fatty acid (coconut oil, lauric acid oil blend, lauric/myristic acid oil blend)<br><br>(*14.2 to 108 g/d)	-Long-chain fatty acid (palm oil, tallow, butter, palmitic acid oil blend ((55% diary fat + 36% palm stearin + 9% sunflower oil), (22–33 g palm oil + 2–5 g soybean oil), (55% palm stearin + 25% palm oil + 10% palm kernel oil + 10% corn oil)).
Schwingshackl et al., 2018 [23]	March 2018	Healthy, hyperlipidemia, peripheral artery disease, high atherosclerotic risk, Hyperfibrinogen, hypertensive, NAFLD, obesity, low HDL patients, NIDDM	20.2-31.1	54 trials (55 reports)	Random-effects network meta-analysis	Soy oil, palm oil, coconut oil, lard	-Direct comparison: corn oil, palm oil, lard, coconut oil, butter, tallow, olive oil<br><br>-Indirect comparison: butter, safflower oil, flaxseed oil, hempseed oil, tallow, sunflower oil, canola oil, safflower oil, corn oil

HL: High linoleic; HO: High oleic; MUFA: monounsaturated fatty acid; NAFLD: Non-alcoholic fatty liver disease; NIDDM: Non-insulin dependent diabetes mellitus; MCFA: Medium chain fatty acid; PHO: Partially hydrogenated oil; PUFA: Polyunsaturated fatty acid; RBO: Rice bran oil; RCT: Randomized controlled trial

Supplementary Table S3: Degree of Overlapping Individual Studies in LDL Outcome

No	Author, Year	Intervention	Comparator	Harland (2009)	Mozaffarian (2009)	Fattore (2014)	Sun (2015)	Jolfaie (2016)	Ghobadi (2018) (Canola)	Ghobadi (2018) (Olive)	Panth (2018)	Schwingshackl (2018)	Number of overlapping
1	Laine et al., 1982	Soybean oil	Corn oil, palm oil		X								1
2	Baudet et al., 1984	Palm oil	Sunflower oil, peanut oil			X						X	2
3	Mattson et al., 1985	Palm oil	HO safflower oil, HL safflower oil			X	X						2
4	Reiser et al., 1985	Coconut oil	Tallow, safflower oil								X	X	2
5	Baudet et al., 1988	Dairy fat	Canola oil						X				1
6	Bonanome et al., 1988	Palmitic acid	Stearic acid, HO safflower oil			X	X						2
7	Raguram et al., 1989	RBO	Palm + peanut oil					X					1
8	Mensink and Katan., 1990	Palm oil	Trans fat, oleic acid diet		X								1
9	Hornstra et al, 1991	Palm oil	Usual oil			x							1
10	Marzuki et al., 1991	Palm oil	Soybean oil			X	X						2
11	Ng et al., 1991	Palm oil	Coconut oil			X	X						2
12	Denke et al., 1992	Palm oil	HO sunflower oil, high-lauric acid, synthetic oil			X	X						2
13	Heber et al., 1992	Palm oil	Coconut oil, hydrogenated soybean oil			X					X		2
14	Nestel et al., 1992	Palmitic acid	Oleic acid, elaidic acid			X							1
15	Ng et al., 1992	Palm oil	Olive oil			X	X					X	3
16	Truswell et al., 1992: 21	Palm oil	Canola				X						1
17	Truswell et al., 1992: 30	Palm oil	Canola				X						1
18	Zock and Katan., 1992	Saturated fat	HL sunflower oil, trans fat		X								1
19	Kris-Etherton et al., 1993	Soybean oil	Olive oil, butter									X	1
20	Seppanen-Laakso et al., 1993	Saturated fat	Canola oil						X				1
21	Wood et al., 1993	Palm oil	HL sunflower oil, Trans fat				X						1
22	Insull et al., 1994	Soybean oil	Corn oil, sunflower oil									X	1
23	Judd et al., 1994	Saturated fat	HO fat, trans fat		X								1
24	Lichtenstein et al., 1994	RBO	Corn oil					X					1
25	Nestel et al., 1994	Palmitoleic acid	Oleic acid, palmitic acid			X							1
26	Sundram et al., 1994	Palmitic acid	Myristic + lauric acid			X					X		2
27	Tholstrup et al., 1994	Palmitic acid	Stearic acid, myristic + lauric acid			X							1
28	Tholstrup et al., 1994	Palmitic acid	Myristic acid			X							1

29	Uusitupa et al., 1994	Saturated fat	Canola oil						X				1
30	Zock et al., 1994	Palm oil	Sunflower oil, myristic acid			X							1
31	Almendingen et al., 1995	PHO soybean oil	PHO fish oil, butter		X								1
32	Choudhury et al., 1995	Palm olein	Olive oil			X	X			X		X	4
33	Cox et al., 1995	Coconut oil	Butter								X		1
34	Ghafoorunissa et al., 1995: 12	Palm olein	Peanut oil			X	X						2
35	Ghafoorunissa et al., 1995: 24	Palm olein	Peanut oil			X	X						2
36	McKenney et al., 1995	Coconut oil	Canola oil						X				1
37	Nestel et al., 1995	High-palm oil margarine	Interesterified high palm oil margarine, HL trans fatty acid margarine			X							1
38	Schwab et al., 1995	Palm oil	Coconut oil			X					X	X	3
39	Sundram et al., 1995	Palm olein	Canola oil, AHA blend			X	X		X			X	4
40	Tholstrup et al., 1995	Palm oil	Myristic and lauric acid, stearic acid			X					X		2
41	Zock et al., 1995	Palm oil	Modified palm oil esterified in sn-2 position			X							1
42	Noakes et al., 1996	Palm oil	HO sunflower oil, oil blend			X	X						2
43	Schwab et al., 1996	Palmitic acid	Stearic acid			X							1
44	Temme et al., 1996	Palmitic acid	Lauric acid, oleic acid			X					X		2
45	Aro et al., 1997	Saturated fat	Trans fat		X								1
46	Cater et al., 1997	Palm oil	HO sunflower oil, MCT oil, synthetic oil			X	X					X	3
47	Choudhury et al., 1997	Palm oil crisps	HO sunflower crisp			X	X						2
48	Storm et al., 1997	Palmitic acid rich	Stearic rich			X							1
49	Sundram et al., 1997	Palm oil	Habitual diet, trans soybean oil, HO mixture			X	X						2
50	Zhang et al., 1997 parallel	Palm oil	Peanut oil			X							1
51	Zhang et al., 1997 cross over	Palm oil	Soybean oil, peanut oil, lard			X	X					X	3
52	Cox et al., 1998	Coconut oil	Butter								X		1
53	Cuesta et al., 1998	Palm olein	HO sunflower oil			X	X						2
54	Judd et al., 1998	Saturated fat	Trans fat		X								1
55	Muller et al., 1998	Palm oil	Trans fat margarine, PUFA margarine		X	X	X						3
56	Nestel et al., 1998	Palmitic acid	Stearic acid			X							1
57	Sarkkinen et al., 1998	Saturated fat	Canola oil						X				1
58	Lichtenstein et al., 1999	Soybean oil	Butter		X							X	2
59	Mutalib et al., 1999	Palm oil	Hydrogenated soya fat, hydrogenated canola oil			X	X						2
60	Rajnarayana et al., 2000	RBO	none					X					1
61	Vissers et al., 2000	RBO	Sunflower oil					X					1
62	Cater et al., 2001	Palm oil	HO sunflower oil				X					X	2

63	de Roos et al., 2001	Lauric acid rich fat	Trans fat		X								1
64	Sondergren et al., 2001	Palm oil	Canola oil	X					X				2
65	Judd et al., 2002	Stearic acid rich fat	HO fat, trans fat		X								1
66	Lovejoy et al., 2002	Palmitic acid	HO fat, trans fat		X								1
67	Bosch et al., 2002	Palm oil	Soybean oil + sunflower oil				X						1
68	French et al., 2002	Palmitic acid	Linoleic acid			X							1
69	Han et al., 2002	Soybean oil	Butter									X	1
70	Kelly et al., 2002	Palmitic acid	Stearic acid			X							1
71	Montoya et al., 2002	Palm oil	Oleic acid, sunflower oil, fish oil			X							1
72	Sanchez-Muniz et al., 2002	Palm olein	Sunflower oil			X	X						2
73	Sundram et al., 2003	High palmitic acid	High trans fat			X	X						2
74	Zhang et al., 2003	Red palm oil	Soybean oil			X							1
75	Scholtz et al., 2004	Red palm oil	Sunflower oil			X	X					X	3
76	Berger et al., 2005	RBO	Peanut oil					X					1
77	Kuriyan et al., 2005	RBO	Sunflower oil					X					1
78	Most et al., 2005	RBO	Oil blend					X					1
79	Pedersen et al., 2005	Palm-margarine (trans-free)	Trans-margarine, PUFA margarine from sunflower oil			X							1
80	Vega-Lopez et al., 2006	Palm oil	Canola oil, soybean oil, partially hydrogenated soybean oil	X		X	X		X				4
81	Forsythe et al., 2007	Palmitic acid	High linoleic acid sn-2			X							1
82	Sundram et al., 2007	Palm olein	Trans-partially hydrogenated soybean oil, Interesterified stearic acid		X	X	X					X	4
83	Mensink et al., 2008	Palmitic acid	HO oil			X	X						2
84	Assuncao et al., 2009	Coconut oil	Soybean oil									X	1
85	Utarwuthipong et al., 2009	Palm oil	Soybean oil, oil blend (rice bran oil + palm oil)			X	X	X				X	4
86	Vega-Lopez et al., 2009	Soybean oil	Corn oil									X	1
87	Teng et al., 2010	Palm stearin	HO palm olein, partially hydrogenated soybean oil			X						X	2
88	Eady, 2011	Rice bran oil	Standard spread					X					1
89	Iggman et al., 2011	Dairy fat diet	Canola oil						X				1
90	Tholstrup et al., 2011	Palm olein	Olive oil, lard			X	X			X		X	4
91	Voon et al., 2011	Palm oil	Olive oil, coconut oil				X				X	X	3
92	Lai et al., 2012	RBO	Soybean oil					X					1
93	Zavoshy et al., 2012	RBO	Low calories diet without RBO					X					1
94	Filippou et al., 2014	Palm oil	Sunflower oil									X	1
95	Iggman et al., 2015	Palm oil	Sunflower oil									X	1
96	Lucci et al., 2016	Palm oil	Virgin olive oil							X		X	2
97	Candido et al., 2017	Soybean oil	Olive oil									X	1
98	Chang et al., 2017	Palm oil	Sunflower oil									X	1

99	Harris et al., 2017	Coconut oil	Safflower oil									X	1
100	Khaw K-T et al., 2018	Coconut oil	Butter, olive oil								X	X	2
	Total (100)			2	13	51	32	11	9	3	10	28	159
%Overlapping					0.400 =40%								
Covered Area (CA)					0.177=17.7%								
Corrected Covered Area (CCA)					0.074=7.4%								

Supplementary Table S4: AMSTARII of Studies with Lipid Outcomes

Author	1.PICO Y/N	2.Deviation Y/P/N	3.Selection of Study	4.Comprehensive literature search Y/P/N	5.Duplicate selection	6.Duplicated data extraction	7.List of excluded studies	8.Describe included studies	9.Bias assessment (RCT/NRSI) Y/P/N	10.Source of individual	11.Adequated statistical method	12.RoB risk of bias in individual	13.Discussion using risk of bias	14.Explain heterogeneity	15.Publication bias assessment	16.Conflict of interest	Interpret
Harland, 2009	Y	N	Y	N	N	N	N	P	N	N	Y	N	N	N	Y	Y	Critically low
Mozaffarian, 2009	Y	N	Y	N	N	N	N	P	N	N	Y	N	N	N	N	Y	Critically low
Fattore, 2014	Y	P	Y	P	Y	N	N	Y	N	Y	Y	N	N	Y	Y	Y	Critically low
Sun, 2015	Y	Y	Y	N	Y	Y	Y	Y	Y	Y	Y	Y	Y	Y	Y	Y	Low
Jolfaie, 2016	Y	N	Y	P	Y	Y	Y	Y	Y	N	Y	Y	Y	Y	Y	Y	Low
Ghobadi, 2018 Canola oil	Y	Y	Y	P	Y	N	Y	Y	Y	N	Y	Y	Y	Y	Y	Y	Moderate
Ghobadi, 2018 Olive oil	Y	Y	Y	P	Y	Y	Y	Y	Y	N	Y	Y	Y	Y	Y	Y	High
Panth, 2018	Y	Y	Y	P	Y	Y	Y	Y	Y	N	Y	Y	Y	Y	Y	Y	High
Schwingshackl, 2018	Y	Y	Y	P	Y	Y	Y	Y	Y	Y	Y	Y	Y	Y	Y	Y	High
